# Electrical Neuroimaging of Music Processing Reveals Mid-Latency Changes with Level of Musical Expertise

**DOI:** 10.3389/fnins.2017.00613

**Published:** 2017-11-07

**Authors:** Clara E. James, Mathias S. Oechslin, Christoph M. Michel, Michael De Pretto

**Affiliations:** ^1^School of Health Sciences, University of Applied Sciences and Arts Western Switzerland, Geneva, Switzerland; ^2^Faculty of Psychology and Educational Sciences, University of Geneva, Geneva, Switzerland; ^3^Neuroscience Center, University of Geneva, Geneva, Switzerland; ^4^Department of Education and Culture of the Canton of Thurgau, Frauenfeld, Switzerland; ^5^Functional Brain Mapping Lab, Department of Fundamental Neurosciences, University of Geneva, Geneva, Switzerland; ^6^Center for Biomedical Imaging (CIBM), Lausanne, Switzerland; ^7^Neurology Unit, Medicine Department, Faculty of Sciences, University of Fribourg, Fribourg, Switzerland

**Keywords:** musical training, musical syntax transgression, microstate analysis, ERP source imaging, middle temporal gyrus, anterior cingulate gyrus

## Abstract

This original research focused on the effect of musical training intensity on cerebral and behavioral processing of complex music using high-density event-related potential (ERP) approaches. Recently we have been able to show progressive changes with training in gray and white matter, and higher order brain functioning using (f)MRI [(functional) Magnetic Resonance Imaging], as well as changes in musical and general cognitive functioning. The current study investigated the same population of non-musicians, amateur pianists and expert pianists using spatio-temporal ERP analysis, by means of microstate analysis, and ERP source imaging. The stimuli consisted of complex musical compositions containing three levels of transgression of musical syntax at closure that participants appraised. ERP waveforms, microstates and underlying brain sources revealed gradual differences according to musical expertise in a 300–500 ms window after the onset of the terminal chords of the pieces. Within this time-window, processing seemed to concern context-based memory updating, indicated by a P3b-like component or microstate for which underlying sources were localized in the right middle temporal gyrus, anterior cingulate and right parahippocampal areas. Given that the 3 expertise groups were carefully matched for demographic factors, these results provide evidence of the progressive impact of training on brain and behavior.

## Introduction

The current research applied high-density EEG (electroencephalography) to investigate the impact of musical training on brain and behavioral processing of complex classical music in non-musicians, amateur pianists, and professional pianists. All pianists were trained in the western classical repertoire.

The musical brain is “food for neuroscience” (Zatorre and McGill, 2005) and, at present, a prevalent way to study functional and structural brain plasticity (Pantev and Herholz, 2011). Several reasons may explain this. First, musical training intensity in the general population is not disparate, but a continuum, going from passive exposure, which already leads to some implicit learning and plasticity (Koelsch et al., 2000; Tillmann et al., 2000; Bigand, 2003), to people spending the major part of their active life training on their musical instrument (Pascual-Leone, 2001; Jancke, 2009; Wan and Schlaug, 2010). Second, presenting rich and complex musical stimuli in neuroimaging settings provides an efficient way to investigate the fruit of musical training. Finally, because musical training is multi-modal and involves practically all human cognitive functions (Pantev and Herholz, 2011; Herholz and Zatorre, 2012), indications are accumulating that the benefits of musical training extend beyond the musical domain (Moreno et al., 2009; Parbery-Clark et al., 2009; Schon and Francois, 2011; Kraus et al., 2012; Barrett et al., 2013; Miendlarzewska and Trost, 2013; Oechslin et al., 2013a,b).

Whether more training leads to enhanced expertise is, of course, a matter of debate; however, until further research is undertaken, the amount of practice is the only valid predictor of performance level in musicians (Ericsson et al., 1993; Sloboda et al., 1996).

The best approach to evaluate the impact of long-term, high-level musical training on brain processing and structure would be a longitudinal one. Several longitudinal studies extending more than 6 months could show the impact on brain structure and function following musical training in children (Hyde et al., 2009a,b; Moreno et al., 2009; Ellis et al., 2013; Habibi et al., 2016). However, following groups of future professional, amateur and non-musicians from childhood over a period of at least 10 years, the minimum time it takes to become an expert musician (Ericsson et al., 1993; Sloboda et al., 1996), is a costly if not impossible operation. A good alternative is a cross sectional study on groups with distinct levels of expertise with strict matching of the different groups (Jancke, 2009). In the series of experiments we conducted (Oechslin et al., 2013a,b, 2017; James et al., 2014; Jenni et al., 2017), we matched 3 groups with different expertise levels for age, gender, education level, age of training onset, training intensity and fluid intelligence, resulting in three rigorously controlled distinct and progressive levels of musical expertise and proficiency. In these studies, we were able to show gradual brain function and structure development with increasing musical expertise and training. The current study is part of that series and investigated the same participant groups.

The crux of our series of studies partially relies on the stimuli used. By conducting behavioral pilot studies and engaging a professional composer and sound engineer, we managed to create expressive realistic musical stimuli containing refined in-key transgressions of musical chord structure or syntax at musical closure. These stimuli allowed us to perfectly separate levels of expertise by means of behavioral appraisal responses. In contrast, the well-known Gordon's AMMA (“Advanced Measures of Music Audiation”; Gordon, 1989) could not do so well. In the AMMA test, participants must detect either tonal or rhythmic differences between pairs of melodies; the tonal subtest taps into processing that is partially similar to our paradigm. Applying AMMA to our population, differences between amateurs and non-musicians did not reach significance for the tonal subtest, only between experts and both other groups, so the intermediate position of the amateurs could not be evidenced (see Figure 6C in Oechslin et al., 2013b). Moreover, using polyphonic realistic musical stimuli in all 24 tonalities and in different classical music styles allows for drawing more general conclusions on the processing of western tonal music.

Here, we used high-density EEG to investigate the influence of musical training on processing of complex music in the exact same population as in our previous (f)MRI studies (Oechslin et al., 2013a,b, 2017; James et al., 2014). Electrical neuroimaging may shed additional light on this issue because it adds information on the time course of brain processing with millisecond precision, accompanied by concurring source localizations. Periods of interest showing a significant effect of experimental manipulations and expertise on brain processing were identified using microstate analysis.

The concept of “microstates” derives from the insight that evoked potentials manifest as stable voltage topographies over time, lasting tens to hundreds of milliseconds, separated by brief periods of transition (Lehmann et al., 1987; Murray et al., 2008; Brunet et al., 2011). These microstates reflect, as their name suggests, “microstates of information processing,” i.e. basic physiological units of cognition (Lehmann et al., 1987), that correspond to a time window of coherent synchronized activation of large-scale neuronal networks (Brunet et al., 2011). Microstate analysis or spatio-temporal ERP analysis enables extracting these stable scalp voltage topographies over time in a multivariate way, comprising all electrodes, all groups and all conditions in one unified analysis, thus conserving the major part of the variance in the data. In contrast, the isolated amplitude analysis of an array of single electrode sites only uses a small part of the variance in the data and moreover ignores the spatial dimension of high-density EEG (Michel and Murray, 2012). In the context of our complex experimental plan, microstate analysis is therefore highly appropriate, especially to analyze processing states of acoustic musical stimuli with high temporal resolution, and compare the underlying brain sources with the results of our earlier fMRI study (Oechslin et al., 2013b). By physical laws, changes of scalp voltage topographies, or microstates, indicate a change in the configuration of the active cerebral sources (Vaughan, 1982; Michel and Murray, 2012). Therefore, microstate analysis is a precursor for EEG source imaging (Michel and Murray, 2012).

Whereas EEG directly measures neural activity with millisecond precision, fMRI measures neural activity indirectly via the BOLD (blood-oxygen-level dependent) response. In consequence, the temporal resolution of fMRI is limited to several seconds (Menon and Crottaz-Herbette, 2005). Although fMRI resolution is an order of magnitude better than that of EEG, with millimeter vs. centimeter precision (Martuzzi et al., 2009; Plomp et al., 2010; Birot et al., 2014), electrical source imaging methods using scalp EEG similar to the ones used in the current study have been successfully applied in different experimental and clinical domains (James et al., 2008, 2015; Britz et al., 2009, 2014; Plomp et al., 2010, 2013; Knebel et al., 2011; Rihs et al., 2013; De Meo et al., 2015). The validity and precision of source localizations based on high-density EEG (more than 100 electrodes), when using sophisticated source and head models, are generally underestimated (Michel et al., 2004; Plomp et al., 2010; Michel and Murray, 2012). Other imaging methods or direct intracranial recordings validated the correctness and precision of these EEG source localizations (Lantz et al., 2001; Tse and Penney, 2008; Megevand et al., 2014; Centeno et al., 2017). Finally, ERP source imaging adds the millisecond range temporal dimension.

There is a linear relationship between stimulus-driven local field potentials and the BOLD response (Logothetis et al., 2001; Martuzzi et al., 2009). EEG and fMRI activations in response to identical experimental stimulations may show both overlap and differences as they reflect different aspects of neuronal activity (Disbrow et al., 2000; Plomp et al., 2010). In a combined EEG-fMRI study on semantical priming (Geukes et al., 2013), EEG source imaging was able to detect specific sources of the N400. In contrast, fMRI reflected a general effect of task in areas comprising the EEG N400 sources. In this study, ERP source imaging appeared more sensitive to subtle cognitive effects. In a face processing task, fMRI and EEG source imaging results also partially overlapped (Corrigan et al., 2009).

In conclusion, our experimental setting allowed to test the following hypotheses (1) ERP analyses will confirm gradual impact of training intensity on musical processing; (2) Given the refined nature of the transgressions, the time window of these differences will reflect later cognitive processing; (3) Specific microstates will appear as a function of expertise and transgression level, evidencing differential processing; (4) The underlying brain sources may partially correspond to those found with fMRI.

## Material and methods

### Participants

The participants of this experiment were the same individuals as in our previous 3 studies (Oechslin et al., 2013a,b; James et al., 2014). All pianists received training in classical music.

EEG was always recorded after fMRI to control for learning effects on the performance of the musical task. By keeping the same order for all participants, we ensured that the amount of practice of the musical task before the EEG session was the same for everyone. We started with the fMRI recording because it included fewer stimuli (*n* = 30) than during the EEG recording (*n* = 90), thus minimizing this learning effect.

The local faculty ethics commission (Commission d'éthique de la Faculté de Psychologie et des Sciences de l'Education de l'Université de Genève) approved the protocol, and 59 right-handed (“Edinburgh Inventory”; Oldfield, 1971) volunteers consented to participate and received financial compensation. All participants reported normal hearing and no history of neurological illnesses. Three groups with distinct training patterns were recruited, and consisted of 19 non-musicians (Non-musicians, 24.0 ± 4.5 years; 9 women), 20 amateur pianists (Amateurs, 22.2 ± 3.1 years; 11 women) and 20 expert pianists (Experts, 24.5 ± 4.5 years; 10 women). No significant differences occurred for gender, age [*F*_(2, 56)_ = 2.2, *p* = 0.12] or fluid intelligence measured by Raven's Advanced Progressive Matrices [*F*_(2, 58)_ = 1.2, *p* = 0.31].

All Non-musicians and Amateurs were academic students, except for one secondary school student and one academic. Experts were principally advanced conservatory students, but a minority were already performing artists and/or teachers; they had received training at the Conservatoires of Geneva, Lausanne, Paris and Zurich. Following our inclusion/exclusion criteria, Non-musicians had not received any extracurricular musical education and had never practiced a musical instrument; Amateurs' practice had never exceeded 10 h of training per week and was not interrupted between childhood and the moment of testing; the latter rule also applied to the Experts. Musicians (Amateurs and Experts) began piano practice before the age of ten. Age of training onset did not significantly differ [*t*_(38)_ = 1.5, *p* = 0.19] between Amateurs (7.0 ± 1.4 years) and Experts (6.2 ± 1.9 years).

Training intensities in different age brackets were assessed by means of a retrospective questionnaire. For the first two age brackets (6–8 and 8–10 years), no significant differences occurred, whereas the subsequent age periods showed a consistently increasing difference between Amateurs and Experts for training hours per week (see Table 1). Levels of expertise were confirmed by distinct behavioral responses from the 3 groups to our musical test [see Experimental procedure and in-house musical test (during EEG)].

**Table 1:** Training intensities of Amateurs (A) and Experts (E).

**Age brackets**	**Hours per week**		***t*-values**
	**A**	**E**	
6–8	3.0 (±1.9)	3.1 (±1.7)	*t*26: 1.1
8–10	3.5 (±0.5)	4.2 (±0.5)	*t*38: 1.1
10–12	4.0 (±2.3)	6.5 (±4.3)	*t*38: 2.7^*^
12–14	4.7 (±2.6)	9.0 (±5.3)	*t*38: 3.3^**^
14–16	5.3 (±3.2)	14.8 (±7.7)	*t*38: 5.1^***^
16–18	4.7 (±2.2)	19.9 (±9.3)	*t*38: 7.1^***^
18–25	4.8 (±2.6)	30.7 (±8.5)	*t*38: 12.4^***^

*Between-group differences assessed by t-tests between E (Experts) and A (Amateurs) at each age bracket*.

* *p < 0.05*,

** *p < 0.01*,

*** *p < 0.001. A, Amateurs; E, Experts. Adapted from James et al. (2014), with permission of Springer*.

### Experimental procedure and in-house musical test (during EEG)

A professional composer and sound engineer (see section Acknowledgements) composed 120 expressive string quartets in classical style for our experiments, with an average duration of 10 seconds, equally distributed over all 24 minor and major tonalities. The expressive pieces varied strongly in character and classical style (from baroque to romanticism). The music was arranged using the Sibelius software (Avid Technology, Inc.) and “Logic Pro” (Apple Inc.); natural instrumental timbres were implemented (Garritan Personal Orchestra). Of this material 30 items served as stimuli for sparse sampling fMRI (Oechslin et al., 2013b), and the remaining 90 for the current EEG study.

**Figure 1:**
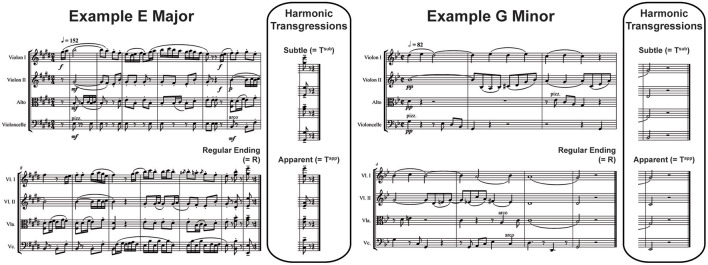
Examples of Stimuli. A composition for string quartet in E major **(Left)** and another in G minor (**Right**; 2 out of 90 in total). Three versions of each were presented, each of which featured a Regular Ending (Tonic chord in root position), a Subtly Transgressed Ending (T^sub^; Tonic chord in first inversion) or an Apparently Transgressed Ending (T^app^; Subdominant in first inversion).

Each of the stimuli appeared in 3 versions, with regular (R), subtly transgressed (T^sub^) and apparently transgressed (T^app^) closure. The harmonic^1^ transgressions were syntactical in nature and consisted respectively of R endings: tonic chords in root position (I), T^sub^ endings: tonic chords in first inversion (I^6^) and T^app^ endings: subdominant or IVth degree chords in first inversion (for an example score, see Figure 1; for the corresponding sound files, see Supplementary Material; for more music examples and comprehensive explanations of the choice of the violations used, refer to Oechslin et al. (2013b) and Jenni et al. (2017).

The concept of musical syntax is now well established and defined as a hierarchical structure hosting nested nonlocal dependencies (Koelsch et al., 2013). However, some controversy legitimately exists regarding the degree of similarity of musical vs. language syntax (Asano and Boeckx, 2015), but this issue is not relevant in the present context, because we only investigated music processing.

The syntactic transgressions used here represent nonlocal dependencies as the expectation of the tonic chord at closure is built up throughout the piece. Thus, the paradigm used is expectation violation.

We converted the original stereo sound files to mono in order to prevent left vs. right ear bias. The terminal chords of all pieces were cut off 1,400 ms after onset and linearly faded over the last 150 ms to create stimuli of identical length (using Adobe Audition 3.0, Adobe Systems Corp., California, USA). All stimuli were normalized to an identical amplitude envelope (-5 dB). Stimuli were presented binaurally with E-Prime 1.0 software (Psychology Software Tools, Pittsburgh, PA) via EEG compatible earphones (Etymotic Research, Elk Grove Village, IL, USA). We do not report mean intensity of stimulus presentation because we let each individual determine optimal loudness. As a function of the size and shape of the ear cup and canal, the insert earphones entered more or less deeply in the ear canal, and the distance to the eardrum strongly influences perceived loudness.

EEG triggers (scripted in milliseconds in E-Prime) indicated the onset of each sound file, the onset of each terminal chord and the ending of each sound file. The onset of the terminal chords (stimulus onset) were automatically marked with Adobe Audition 3.0 using a burst criterion “find beats” if an increase of minimum 8 dB occurred within 14 ms (best criterion for our stimuli). Bursts were then verified one by one by eyeballing the triggers in the magnified sound files while listening to the stimuli. Synchronization of EEG recordings and stimulus presentation by E-Prime (i.e., correct placement of the triggers in the EEG recordings) was certified by comparing the simultaneous sound output and EEG output. For that purpose, we connected the sound output plug (to be plugged into the earphones) with a subset of the electrodes in a conductive medium; differences were kept below 5 ms, by means of corrections of the E-prime script.

Identical written task explanations were provided to all participants, independently of expertise level, before the experiment. Once the EEG material mounted, three example stimuli that did not occur in the test were appraised in the final EEG setting, just before starting the recordings. If necessary, instructions were explained again using the same terminology for all three groups, avoiding musical jargon in order so as not to benefit the musicians. To prevent facilitation and biasing of the task, we did not inform the participants about the double nature of the transgressions. Participants responded YES if they appraised the stimuli as “satisfactory” or “correct” and NO otherwise. Correct answers were thus YES in response to R and NO in response to both T^sub^ and T^app^. They responded by means of pressing buttons with the index and middle finger of the right hand on a button box. To prevent motor contamination of the EEG signal, participants were asked to postpone their response until a prompt (“please respond”) appeared on the computer screen, 1,900 ms after the onset of the terminal chord of the musical piece. Therefore, we do not report reaction times.

Participants were installed in a cabin with electromagnetic and sound shielding, in a comfortable chair, and eye-screen distance was around 1 m. The stimuli (*n* = 3^*^90) were presented in 5 blocks, separated by breaks. Because the total duration of the presentation of all stimuli was approximately one hour without breaks, the participants were in control of launching each stimulus, enabling them to take brief pauses during the blocks. With this approach, we prevented fatigue and movement during stimulus presentation. Before beginning the experiment, we explained to the participants that their movement and muscular tension would compromise the EEG signal and showed them the EEG signal while they moved, blinked, etc. We encouraged them to blink, stretch, yawn and change their position in between the stimuli. Then, we asked them to avoid blinking, movement or muscle tension after the music ended until the prompt (“please respond”) appeared. During presentation of the stimuli, participants looked at a fixation cross in the middle of a light gray computer screen to prevent eye strain; they received the instruction not to close their eyes while the music played. The order of the stimuli was pseudo-randomized, preventing different versions of the same stimulus being presented in succession. To avoid sequential effects, a version with reversed order of all stimuli was used in alternation for half of the subjects in each group.

First, we computed the percent correct for the binary responses (YES/NO) to R, T^sub^ and T^app^ endings. Based on these binary results, we computed d-prime values for T^sub^ and T^app^. The d-prime parameter derives from signal detection theory (Macmillan and Creelman, 1997) and prevents impact of response bias, taking into account false alarms, i.e., “NO” responses to R endings. D-prime represents sensitivity to the transgressions: Higher d-prime values reflect better discrimination between transgressed and regular endings; negative d-prime values, which indicate a lack of sensitivity to the transgressions, may occur if more false alarms occur than “hits” (correct detection of transgression, NO in response to T^sub^ or T^app^). Finally, a 2-way ANOVA comparing d-prime values for T^sub^ and T^app^ for all groups was performed (see Results for specification of the ANOVA), and pertinent Bonferroni corrected contrasts were computed.

### EEG acquisition and raw data processing

Continuous EEG was recorded, with vertex reference, at a sampling rate of 1,000 Hz at 256 electrode sites with a HydroCel Geodesic Sensor Net (Electrical Geodesics Inc., Eugene, OR, USA).

Measuring the inion-nasion distance for each individual allowed us to place the electrode net in such a way that equivalents of midline electrodes Fz, Cz (the vertex) and Pz corresponded to the international 10/10 system (Luu and Ferree, 2005). The tension structure and the choice of the size of the net warranted even distribution of all electrodes over the scalp, at uniform positions across participants.

We kept impedances below 30 Ω. We excluded the sensors at the cheeks and in the lower neck that are prone to artifacts and only used the remaining 204 electrodes for analysis (Rihs et al., 2013; Britz et al., 2014). After recording, data were average-referenced, band-pass filtered (0.25–30 Hz) with a 2nd-order Butterworth filter (−12 dB/octave roll-off; Brunet et al., 2011), and down-sampled to 250 Hz to speed up data analyses. Noisy channels were interpolated (4.7% ± 2.2 of all channels) with a 3D spherical spline (Perrin et al., 1989; Brunet et al., 2011). We removed artifacts by means of a combination of an automated threshold criterion (100 μV) and visual inspection for oculomotor and other artifacts of all individual epochs (Michel and Brandeis, 2009; Brunet et al., 2011). On average, 70 ± 8 (out of 90) artifact-free epochs per condition and per group were averaged into ERPs from −200 up to 750 ms after stimulus onset (onset of the terminal chord of the musical piece). For waveform analyses, ERPs were baseline corrected (“pre-stimulus” baseline from −200 ms to stimulus onset) to enable comparison to the literature. This data processing was performed with the Cartool software, developed by Denis Brunet (brainmapping.unige.ch/cartool).

### ERP analyses

We analyzed the ERPs in 3 consecutive steps

#### Step 1: ERP waveform analyses

We performed waveform analyses on 2 midline electrodes, Fz and Cz. First, these 2 electrodes are the most frequently used sites for auditory evoked potentials because sources in the Sylvian sulcus project maximally to the top of the head (Hall, 2007). Second, the P300 auditory evoked potential in response to deviant stimuli is classically reported at Fz and Cz (Duarte et al., 2009). In addition, in studies that reported the ERAN (early right anterior negativity) in response to musical syntactic transgressions, this component clearly manifested at Fz and Cz (Koelsch et al., 2001, 2007). Finally, because we used the Geodesic 256 Sensor Net, Cz and Fz are among the most reliable to be placed according to the 10/10 system. To compare the 3 expertise levels for all transgression conditions, we computed Bonferroni corrected contrasts (*t*-tests) for independent samples on mean voltage amplitude over time between grand-average ERPs of Non-musicians, Amateurs and Experts, over the full −200 to 750 ms window, applying a minimum time constraint of 28 consecutive milliseconds (7 time frames at 250 Hz) for all 3 transgression conditions at both electrodes.

Because our main goal for ERP analyses consisted of microstate analyses comprising all electrodes (see Step 2 for an explanation) as a function of expertise level (EX) and syntactic transgression (T), the waveform analyses principally served to enable comparison to the literature.

#### Step 2: microstate analyses

The insight that spontaneous EEG and evoked potentials manifest over time as stable voltage topographies lasting tens to hundreds of milliseconds, separated by short periods of transition, led to the concept of “microstates” (Lehmann et al., 1987; Murray et al., 2008; Brunet et al., 2011). Microstate analysis enables studying these stable scalp voltage topographies over time, comprising all recorded electrodes of different groups and conditions in one unified analysis, resulting in a more global assessment than with classical ERP waveform analyses. Such a multivariate approach is particularly suited in the context of a complex experimental design, such as in the current study, with 3 groups, 3 experimental conditions and 204 electrodes. Indeed, it condenses a great amount of information into one series of microstates, while conserving a maximum of the variance present in the data. One of the benefits of this approach is that these different microstates can be compared between groups and conditions in a multifactorial manner.

In the first stage, a *k*-means cluster analysis on topographic dissimilarity served to segment the grand-mean ERP data of all 204 electrodes of all 3 groups and all 3 transgression conditions over time into one series of *k* microstates or scalp topographies (brainmapping.unige.ch/cartool). The time frame of analysis extended from 0 to 750 ms after stimulus onset (onset of the terminal chord of the musical piece). Topographic dissimilarity in the context of surface EEG is an indicator of spatial difference between 2 voltage scalp configurations (Murray et al., 2008). An optimal choice of *k* is essential in order to build meaningful clusters (Fischer, 2011). For that matter, we used cross-validation criteria, minimizing residual variance, as well as a modified Krzanowski–Lai criterion, which provides a quality measure for each clustering (Krzanowski and Lai, 1988; Pascual-Marqui et al., 1995; Tibshirani and Walther, 2005; Murray et al., 2008; Michel and Brandeis, 2009; Brunet et al., 2011). This approach generated an a priori hypothesis in the form of a microstate series occurring over time, based on this multivariate analysis of grand mean ERPs of all groups and conditions.

In the second stage, this a priori hypothesis on the appearance of microstates over time was statistically verified by means of a procedure that fits the microstates to the time series of each individual over a certain time period during which differences occur (Murray et al., 2008; Brunet et al., 2011). The result consists of measures of microstate presence or “duration” (in milliseconds) that can be statistically compared between groups and conditions. Moreover, such an analysis of changes in scalp voltage topographies directly informs on underlying generators in the brain. By physical laws, changes of scalp voltage topographies indicate a change of underlying generators (Vaughan, 1982). Therefore, the time periods during which different microstates appear simultaneously in different groups or conditions provide us with proper time windows to estimate and compare neuronal generators between groups or conditions. We only retained microstates manifesting over periods longer than 28 ms (7 time frames at 250 Hz) because processing of cognitive information in time intervals shorter than several tens of milliseconds is not plausible (Lehmann et al., 1987; Koenig et al., 2002; Brunet et al., 2011).

Microstate durations compared over conditions and groups are commonly not normally distributed. For example, in a certain group, a certain microstate may occur only sporadically, and in some individuals, it may not occur at all, which may constitute simply an experimental effect because a specific stage of information processing may strongly differ as a function of expertise level or transgression condition. Therefore, we applied nonparametric analyses (Kruskal-Wallis, one-way ANOVAs on ranks and Mann-Whitney *U*-tests).

#### Step 3: electrical source imaging

Because the inverse problem is underdetermined with scalp EEG (multiple solutions would be possible), a priori constraints are necessary to reduce this dimensionality (Michel et al., 2004; Grech et al., 2008). Distinct microstates that occur simultaneously in different groups or experimental conditions, if statistically verified, provide such a priori constraints. We therefore performed the estimation and statistical comparison of underlying neuronal generators over a time window during which significant differences occurred in the presence (duration in milliseconds) of the microstates between the different groups and conditions. Refer to the Results section for precise information on this time window.

The electrical source imaging analysis comprised 2 stages.

In the first stage, we estimated the intracranial current density (CD) distribution (in micro amperes per cubic millimeter; μA/mm^3^) for the evoked potential of each participant and condition, averaged over a time window that manifested strong microstate differences as a function of expertise level (EX) and syntactic transgression (T), deriving from the second stage of the microstate analyses (see Results section). These source estimations were computed from averaged ERP waveforms over this time window at all 204 electrodes into a solution space represented by a 3D grid composed of 3005 nodes. These 3005 nodes were selected from a 6 × 6 × 6 mm grid equally distributed over the gray matter of the average brain provided by the Montreal Neurological Institute.

From these average ERPs expressed in microvolts (μV), we computed the CD (μA/mm^3^) in each node of the 3005 3D grid solution space, using a depth-weighted minimum norm (WMN) distributed linear inverse solution (Hamalainen and Ilmoniemi, 1994; Michel et al., 2004). After applying a homogeneous transformation operation to the volume that rendered it to the best fitting sphere (Spherical Model with Anatomical Constraints, SMAC; Spinelli et al., 2000) we used a 3-shell spherical head model to compute the lead field for the 204 electrodes and the inverse solution based on the WMN constraint.

In the second stage, we compared these brain sources by means of statistical parametric mapping (SPM), i.e., comparing the CD for each voxel/node of the 3D grid of the brain between groups and conditions. We computed an ANOVA EX (3, inter) × T (3, intra) at all nodes of the 3D-grid representing the grey matter of the brain using the Statistical Toolbox for Electrical Neuroimaging (STEN; http://www.unil.ch/line/en/home/menuinst/about-the-line/software--analysis-tools.html). To cope with multiple comparisons, only globular clusters composed of minimum 10 contiguous nodes with an individual *p*-value of < 0.005 met our significance criterion (Knebel et al., 2011; De Meo et al., 2015). Moreover, the spatial criterion of 10 contiguous nodes was verified using the AlphaSim program (http://afni.nimh.nih.gov) under the assumption of a spatial smoothing of 7 mm FWMH (full width at half maximum) and a maximum cluster connection radius of 10 mm in combination with the node p-value of < 0.005 that we applied. After 10,000 Monte Carlo iterations, globular clusters of 10 nodes appeared with a cluster-level likelihood of *p* < 0.001, and a corresponding node-level *p*-value of *p* < 0.00001. Our significance criterion may therefore be considered conservative.

### Additional behavioral measures

In order to verify the effects of general cognition, we also performed a 3-back visual working memory (WM) task on letters (Ludwig et al., 2008) and measured fluid intelligence using Raven's Advanced Progressive Matrices (Raven and Court, 2003 updated 2004). Because these tests were also used in the study by Oechslin et al. (2013b), refer to this communication for details on their features.

## Results

### Behavioral results of the in-house musical test (during EEG)

**Figure 2:**
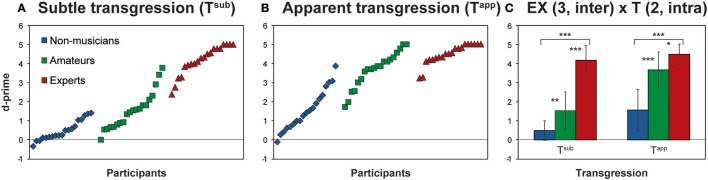
Behavioral Results. **(A,B)** Show individual d-prime scores of all participants for T^sub^ and T^app^, according to Expertise group; **(C)** depicts the mean d-prime scores for each group for both transgression conditions. The main effects of the ANOVA are provided in Table 2. Asterisks indicate Bonferroni corrected contrasts between the groups. ^*^p < 0.05, ^**^p < 0.01, ^***^p < 0.001.

First, we performed a 2-way ANOVA on d-prime for T^app^ and T^sub^ with the factors Expertise (EX) and Transgression (T): EX (3, inter) × T (2, intra). The results of this ANOVA, with main effects for EX and T, as well as interaction EX × T, are reported in Table 2. Both main effects and the interaction effect yielded *p*-values < 0.0001. Figure 2 depicts these results, in Figures 2A,B by displaying all individual d-prime scores for T^sub^ and T^app^, and in Figure 2C, by displaying histograms representing d-prime group means for both transgression conditions.

**Table 2:** ANOVA results on d-prime: EX (3, inter) × T (2, intra).

**Effect**	**df**	**Partial η^2^**	***F*-values**
Expertise	2	0.77	94.7^****^
Transgression	1	0.74	155.8^****^
Expertise × Transgression	2	0.53	31.2^****^

*p < 0.05, **p < 0.01, ***p < 0.001,

**** *p < 0.0001*.

Main effects for both EX and T are increased d-prime values, with expertise and prominence of transgression, respectively. Main effect contrasts were verified by means of Bonferroni corrected t-tests for independent samples between the groups and depicted in Figure 2C; asterisks represent significant *p*-values. For both T^app^ and T^sub^ all comparisons between the groups reached significance.

Hardly any negative d-prime values occurred, showing that almost all participants were sensitive to the transgressions to some extent. Also Non-musicians displayed, albeit moderately, positive d-prime scores in response to both transgressions (3 negative scores on 19 for T^sub^, mean score 0.49 ± 0.52; a single negative score for T^app^, mean score 1.57 ± 1.09; cf. Figures 2A,B).

The interaction effect indicated that for T^app^ and T^sub^ the effect of expertise manifested differently. To check for the exact nature of the interaction effect, we computed simple effects by comparing d-prime values for T^app^ and T^sub^ for each group by means of Bonferroni corrected *t*-tests for dependent samples. Differences for Amateurs [*t*_(19)_ = 10.84, *p* < 0.001; mean T^sub^ = 1.54, mean T^app^ = 3.68] and Non-musicians [*t*_(18)_ = 7.23, *p* < 0.001; mean T^sub^ = 0.49, mean T^app^ = 1.60] reached significance, with higher values for both groups in response to T^app^ than to T^sub^. Responses from Experts did not differ significantly between T^app^ and T^sub^ and were at ceiling for both.

Although, the behavioral results of our in-house musical test are very similar to those from Oechslin et al. (2013b), the results here are slightly different, insofar as all hierarchical contrasts between the groups (Experts > Amateurs > Non-musicians) reached significance for both transgression conditions, whereas in Oechslin et al. (2013b), for T^app^, responses from Amateurs and Experts were not significantly different. Contrasts comparing the EEG vs. fMRI d-prime score for all groups in response to T^app^ confirmed that for Experts only, the d-prime score for the musical test during EEG was higher than during the preceding fMRI [Experts: *t*_(19)_ = 2.59, *p* = 0.018; Amateurs: *t*_(19)_ = 1.37, *p* = 0.188, Non-musicians: *t*_(18)_ = 0.68, *p* = 0.508]. Therefore, apparently, only in Experts, a learning effect took place during the fMRI session that affected d-prime scores collected during EEG.

### EEG analyses

#### Step 1: ERP waveform analyses

**Figure 3:**
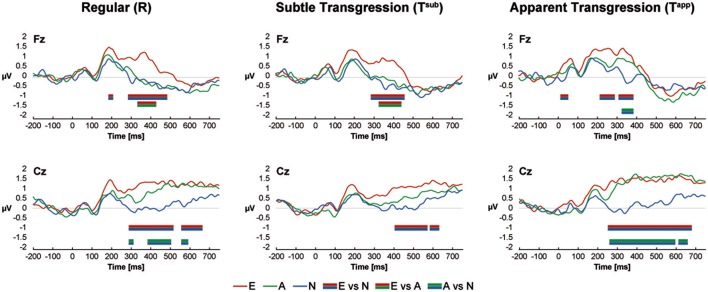
ERP Results at Fz and Cz. Color-coded bars (see legend at the bottom of the graphs) indicate Bonferroni corrected contrasts between the Expertise groups for each Transgression Condition, with a minimum time constraint of 28 consecutive ms.

The ERP curves for all groups and conditions are shown in Figure 3 for the central electrodes Fz and Cz. Color-coded bars represent significant Bonferroni corrected contrasts over time between grand-average ERPs of Non-musicians, Amateurs and Experts, with a minimum time constraint of 28 consecutive milliseconds for all 3 transgression conditions for both electrodes. Differences occurred between approximately 200 and 600 ms after stimulus onset but most often between 300 and 500 ms. Experts systematically displayed more positive voltages, but in response to T^app^, Amateurs' waveforms overlapped those of the Experts at Cz. These positive waveforms are reminiscent of extended P300/P3b ERPs. Interestingly, these results resemble the behavioral d-prime values, showing the largest differences between Experts vs. Non-musicians and Amateurs for T^sub^ and between Experts and Amateurs vs Non-musicians for T^app^. The exact time periods of significant differences are provided in Supplementary Table 1.

#### Step 2: microstate analyses

A k-means cluster analysis yielded 7 ERP microstates that together explained 92.75% of the ERP data variance for all groups and conditions.

**Figure 4:**
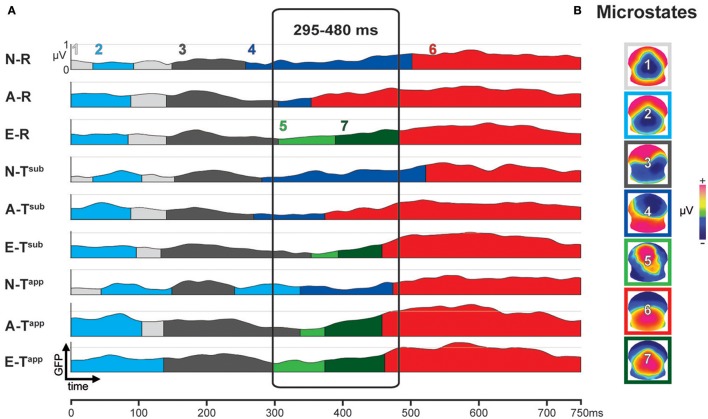
Results of spatio-temporal ERP analysis: functional microstates. **(A)** Color-coded segments below the GFP curve represent the periods of strongest occurrence of the microstates (1–7) in the grand means. **(B)** Scalp potential topographies of ERP microstates, framed in corresponding color-code. N-R, Non-musicians, Regular Endings; A-R, Amateurs, Regular Endings; E-R, Experts, Regular Endings; N-T^sub^, Non-musicians, Subtly Transgressed Endings; A-T^sub^, Amateurs, Subtly Transgressed Endings; E-T^sub^, Experts, Subtly Transgressed Endings; N-T^app^, Non-musicians, Apparently Transgressed Endings; A-T^app^, Amateurs, Apparently Transgressed Endings; E-T^app^, Experts, Apparently Transgressed Endings. GFP, global field power.

Figure 4A depicts the results of the first stage of the microstate analyses. The occurrence of the microstates over time is represented by color coded segments below the 9 global field power (GFP) curves, one for each condition and group. GFP provides a single, positive, reference free value of neural response strength all over the brain by taking into account the absolute voltages of all electrodes (Murray et al., 2008). On the right, Figure 4B shows the scalp potential topographies of the 7 microstates, framed by color codes corresponding to panel A.

**Figure 5:**
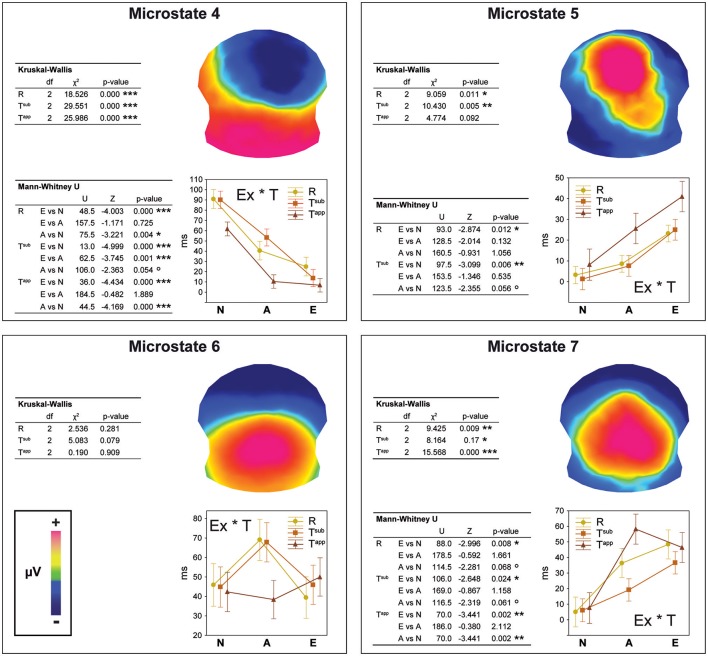
Results of the fitting procedure. Mean duration (in ms) of microstates 4, 5, 6, and 7 compared by means of Kruskal-Wallis ANOVAs between Expertise groups and Transgression conditions for the 295–480 ms period deriving from the spatio-temporal ERP analysis (Figure 4). Additional Bonferroni corrected contrasts using Mann-Whitney *U*-tests are shown in the left bottom table for each microstate. The Expertise groups for each Transgression Condition are compared in the case of significant ANOVA results only.

Between 295 and 480 ms, strong differences occurred between microstates as a function of expertise level and transgression level (cf. Figure 4A). These observed differences were statistically tested in the second stage, comparing the duration (in milliseconds) of Microstates 4, 5, 6, and 7 between all 59 individuals as a function of group and condition. The results of this statistical fitting procedure can be found in Figure 5.

For each microstate, we performed Kruskal-Wallis one-way ANOVAs on ranks to evaluate whether significant differences occurred between the three expertise groups, which were run separately for each of the three conditions (R, T^sub^, and T^app^). In the case of significant ANOVA results only, we performed Bonferroni corrected Mann-Whitney *U*-tests to systematically explore differences between expertise levels. We present the results of these tests in the panels of Figure 5 in a hierarchical way (Experts vs. Amateurs vs. Non-musicians), corresponding to our hypothesis on gradual change with expertise level.

Analyses of durations of Microstates 4 and 7 yielded significant differences for all 3 transgression conditions (R, T^sub^, and T^app^). All of these differences were gradual changes as a function of expertise level, with systematic significant or marginal differences for Experts vs. Non-musicians and Amateurs vs. Non-musicians. Moreover, for Microstate 4, a highly significant difference between Experts and Amateurs occurred for T^sub^. Microstate 4 clearly represents non-musician and amateur processing, showing the longest durations for Non-musicians, intermediate durations for Amateurs, and minimal durations for Experts. Microstate 7 shows the opposite, although Amateurs and Experts showed similar durations for T^app^. Microstate 5, which yielded duration differences between expertise levels for R and T^sub^ reflects expertise level, with the longest average durations for Experts. Although mean duration for T^app^ increased gradually with expertise level for Microstate 5, this difference was not statistically significant due to large variance. Durations for Microstate 6, which seems to be an amateur-specific processing stage based on the fact that Amateurs had the highest values in response to R and T^sub^ endings, did not yield any significant differences.

The overall conclusion is that for maps 4, 5, and 7, gradual differences manifested as a function of expertise, with Non-musicians showing the highest values for Microstate 4, and Experts showing the highest values for Microstates 5 and 7. These “expert” microstates are centro-parietal positivities with extended P300/P3b-like configurations.

#### Step 3: electrical source imaging

Within the time period of significant difference determined in stage 2 of the microstate analyses (295–480 ms), we performed an ANOVA EX (3, inter) × T (3, intra) on CD for all nodes of the 3D grid, which were labeled according to the Talairach atlas. The results are provided in Table 3. For the statistical thresholds used, we refer to the methods section. The *F*-values and Talairach coordinates in the table correspond to the local maxima of each listed area.

**Table 3:** Results of the ANOVA EX (3, inter) × T (3, intra) on current density.

**Brain area**	**RH/LH**	**Talairach**			**Cluster size**	***F*-values**
		**x**	**y**	**z**		
**EXPERTISE** × **TRANSGRESSION**						
Middle Temporal Gyrus	R	43	−67	17	17	5.35
**EXPERTISE**						
Anterior Cingulate	R	9	45	3	13	7.51
Medial Frontal Gyrus	R	9	51	9	19	7.19
Anterior Cingulate	L	−3	38	−1	12	7.14
Medial Frontal Gyrus	L	−3	39	−13	15	6.84
Parahippocampal Gyrus	R	26	−23	−20	42	6.82
**TRANSGRESSION**						
Anterior Cingulate	R	3	38	−7	14	18.97
Medial Frontal Gyrus	L	−3	39	−13	32	18.75
Medial Frontal Gyrus	R	3	39	−13	24	18.71
Anterior Cingulate	L	−3	38	−7	23	18.61
Inferior Frontal Gyrus	L	−16	32	−18	50	16.84
Inferior Frontal Gyrus	R	16	26	−18	22	15.42
Middle Frontal Gyrus	L	−22	32	−12	39	15.04
Parahippocampal Gyrus	L	−20	0	−25	58	14.92
Superior Temporal Gyrus	L	−26	11	−31	24	14.75
Posterior Middle Temporal Gyrus	L	−32	5	−36	10	12.93
Inferior Temporal Gyrus	L	−32	0	−35	11	12.58
Superior Frontal Gyrus	L	−15	63	−8	25	12.45
Insula	L	−43	−11	9	64	12.40
Superior Temporal Gyrus	R	34	19	−28	48	11.74
Insula	R	31	22	4	26	11.61
Parahippocampal Gyrus	R	26	5	−26	40	11.07
Posterior Middle Temporal Gyrus	R	40	6	−27	37	10.45
Inferior Parietal Lobule	R	55	−44	44	65	9.65
Cuneus	R	9	−69	18	17	9.28
Posterior Cingulate	R	20	−63	12	17	9.15
Inferior Temporal Gyrus	R	32	0	−35	11	8.92
Cuneus	L	−3	−70	12	16	7.98
Cingulate Gyrus	R	9	26	27	16	7.62
Superior Parietal Lobule	R	40	−58	52	15	7.60
Cingulate Gyrus	L	−8	7	43	30	7.51
Postcentral Gyrus	R	33	−33	52	14	7.25
Precuneus	R	15	−63	23	11	7.21
Precentral Gyrus	R	33	−21	62	10	7.01

*RH: Right Hemisphere; LH: Left Hemisphere*.

**Figure 6:**
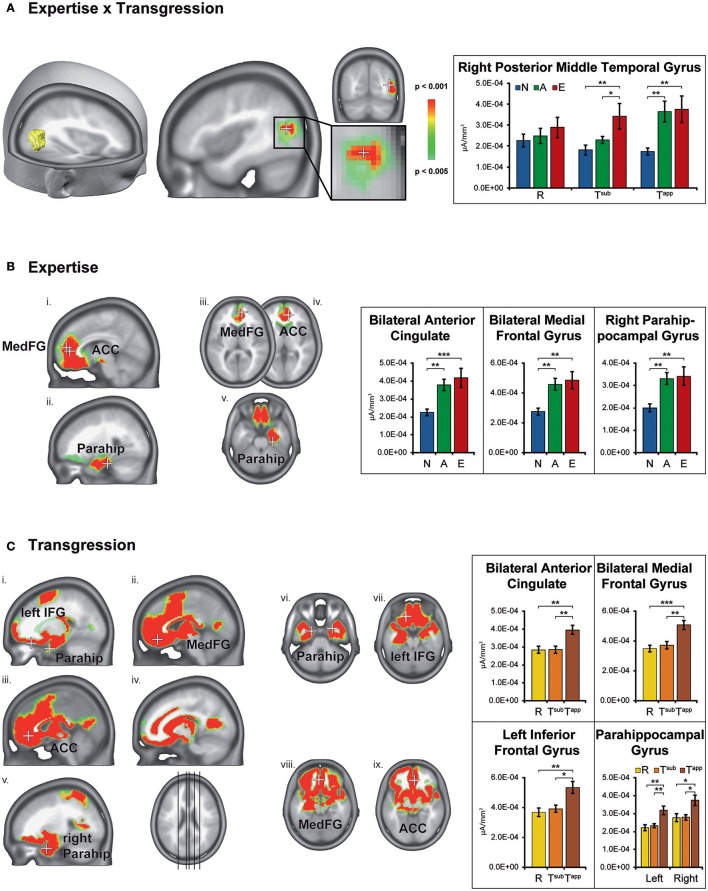
Statistical parametric mapping of distributed electrical source estimations over the time-period after stimulus onset resulting from the microstate analyses (295–480 ms). Results are depicted for Expertise × Transgression interaction **(A)**, main effect of Expertise **(B)**, and main effect of Transgression **(C)**. The bar graphs on the right depict the current densities of the peakvoxels indicated on the left by means of white crosses. N, Non-musicians; A, Amateurs; E, Experts; R, Regular; Tsub, Subtle transgression; Tapp, Apparent transgression. Asterisks indicate LSD-corrected contrasts between the groups. ^*^p < 0.05, ^**^p < 0.01, ^***^p < 0.001.

Figure 6 displays the brain clusters showing significant Expertise × Transgression interaction (Figure 6A), main effects of Expertise (Figure 6B), and main effects of Transgression (Figure 6C), with bar graphs representing the associated CD for the peakvoxel of each Talairach label. Because the main effect of Transgression yielded widely distributed clusters, we only displayed bar graphs for a subset of representative areas, after noting that all brain areas showed a similar CD pattern as a function of transgression condition, i.e., stronger CD values for T^app^ as compared to R and T^sub.^ In order to explore the directionality of the effects, we computed post-hoc analyses using Fisher's least significant difference (LSD) correction for multiple comparisons. Fishers' LSD restricts family-wise error rate to alpha in the special case of 3 groups (Howell, 2014; chapter 8). Significant results of these contrasts are shown by asterisks in the bar graphs in Figure 6.

The Expertise × Transgression interaction yielded differences exclusively in a cluster within the right posterior middle temporal gyrus, close to the lateral occipital complex. The peakvoxel occurred in Brodmann area (BA) 37 (rMTG, Talairach 43, −67, 17, see Figure 6A). No significant differences between the 3 groups occurred for R endings (although a gradual increase of mean CD can be clearly perceived). The *post hoc* tests indicated stronger CD in this brain area in Experts than in Non-musicians and Amateurs in response to T^sub^. In contrast, in response to T^app^, the *post hoc* tests evidenced stronger CD in both Experts and Amateurs as compared to Non-musicians. In other words, T^sub^ yielded stronger CD in experts than in all non-experts, and T^app^ induced stronger CD in all musicians than in non-musicians.

The main effect of Expertise indicated differences within clusters in the bilateral anterior cingulate gyrus, medial frontal gyrus and right parahippocampal gyrus. In all these brain areas, the post-hoc tests displayed stronger CD for Amateurs and Experts than for Non-musicians. In the ACC cluster identified by our analyses, mainly rostral but also dorsal parts of the ACC are present (see Figure 6Bi). The cluster in the right parahippocampal gyrus (see Figure 6Bii) also contained solution points in the amygdala and in the hippocampus proper.

The main effect of Transgression yielded differences in widely distributed brain clusters, including, in order of decreasing *F*-values, the bilateral anterior cingulate gyrus and medial frontal gyrus (see Figures 6Cii,iii,viii,ix), inferior frontal gyrus (see Figures 6Ci,vii), and parahippocampal gyrus (see Figures 6Cv,vi). In these brain areas and all others, the *post hoc* tests indicated stronger CD for T^app^ than for both R and T^sub^. The enhanced activations in response to transgression salience in the bilateral inferior frontal cortex clusters (BA 47, see Figures 6Ci,vii) are situated in the vicinity of the Broca area (BA 44 & 45) and its right hemisphere homolog.

### Additional behavioral measures

In this section, we solely repeat earlier reported findings from Oechslin et al. (2013b). For a visual WM n-back task on letters (Ludwig et al., 2008), we performed Bonferroni corrected *t*-tests for independent samples. Experts (mean = 84.4 [SD = 7.1]) scored significantly better than Non-musicians [mean = 72.1 [SD = 19.7]; *t*_(38)_ = 2.6, *p* < 0.05], but no significant differences occurred with Amateurs (mean = 75.8 [SD = 19]), who held intermediate scores (see Figure 6A in Oechslin et al., 2013b).

For the fluid intelligence test, the Raven's Advanced Progressive Matrices, we did not find any significant differences between the groups.

## Discussion

The key result of this study resides in the gradual influence of musical training and proficiency on processing of musical transgression at the behavioral, ERP, microstate, and source analyses levels. This confirms earlier fMRI, EEG and structural brain data results of the same population (Oechslin et al., 2013b, 2017; James et al., 2014; Jenni et al., 2017). Given the very strict inclusion and exclusion criteria we used, matching for gender, age, age of training onset, instrument practiced, education level and fluid intelligence, and controlling for number of practice hours, we can draw some concrete conclusions on the progressive impact of piano training on brain and behavior. Compellingly, all brain data resembled the pattern of the behavioral d-prime responses of our in-house music test, with the strongest differences occurring between Experts and non-experts (Non-musicians and Amateurs) for responses to subtle transgressions (T^sub^), and between all musicians (Amateurs and Experts) and Non-musicians for responses to apparent transgressions (T^app^).

All EEG, waveform, microstate, and source imaging results that characterized expertise, which was expressed in a mid-latency time window, seem to relate to context-based memory updating. During processing, the actual musical closure is compared with an internal learned model of musical expectancy, which is reinforced with increased training and proficiency. Processing expresses on the waveform level as a P3b-like positivity and on the microstate level as stable centro-parietal positivities with extended P300/P3b like configurations (microstates 5 & 7). These scalp surface configurations are supported at the source level by the right posterior middle temporal gyrus, the bilateral anterior cingulate cortex, as well as by the right parahippocampal complex. In addition, working memory is enhanced in experts.

### Behavioral results of the in-house musical test (during EEG)

Altogether, our in-house test on musical harmony and syntax transgression perfectly separated the groups on the behavioral level, confirming that different levels of musical training intensity yield corresponding levels of musical proficiency, thus mutually validating the composition of our groups and the sensitivity of the in-house musical test.

The crux of the 2-fold transgression detection used here resides in the presentation of subtle and apparent transgressions that are both in-key and do not contain any sensory dissonances as compared to the regular endings. Therefore, they both represent refined transgressions of musical syntax. Such chords would make perfect sense if the music continued, but as an ending they violate the rule system of Western tonal music, which only allows a tonic chord in root position (with the first note of the scale in the bass) at closure. Although the d-prime values for both T^sub^ and T^app^ in Figure 2C gave rise to significant differences between all 3 expertise levels, the Expertise × Transgression interaction effect most likely resulted from the distinct sensitivity of Experts vs. non-experts for T^sub^, and of musicians vs. Non-musicians for T^app^. Indeed, we can clearly observe that for T^sub^, Experts were by far more sensitive; i.e., they display much higher d-prime scores than non-experts (i.e., both Non-musicians and Amateurs), and for T^app^, the d-prime values of both musician/pianist groups (Experts and Amateurs) are relatively close and both groups are very proficient (see Figure 2C). In our fMRI study (Oechslin et al., 2013b), during which we presented one third of the quantity of stimuli presented here, the difference between Amateurs and Experts for T^app^ did not reach significance. The fact that differences between Amateurs and Experts also reached significance for T^app^ in the current EEG study can be explained by the fact that three times as many stimuli were used for the EEG part, providing higher statistical power, but also by a test-retest or learning effect that manifested for T^app^ in Experts only (see section Results). The professionals apparently comprehensively captured the exact nature of the transgressions during the preceding fMRI session, as evidenced by their post-hoc accurate oral testimonies.

In summary, moderate training is sufficient to detect apparent transgressions very well. In contrast, only Experts rated at ceiling the very subtly transgressed T^sub^ endings (tonic chords in first inversion). In the case of dissonant or out-of-key chords, the Non-musicians would probably have rated them equally well as Amateurs or even Experts, as “non-musicians are musical” (Koelsch et al., 2000). However, because the Non-musicians grew up in a context of western tonal music, and therefore implicitly learned the western tonal rule system, most of the Non-musicians exhibited modestly positive d-prime scores for T^sub^, expressing a certain degree of sensitivity to the transgressions. Furthermore, it is highly interesting to observe that the “best” Non-musicians reached the d-prime level of the “worst” Experts for T^app^ (Figure 2B). In other terms, the final performance results are like in most domains the fruit of the interplay between nature and nurture for individuals of all expertise levels.

### Additional behavioral measures

In Oechslin et al. (2013b) we already extensively discussed the benefit of musical training at expert level for visual working memory (WM).

Practicing or performing music is highly demanding concerning WM in the auditory, as well as the visual domain to prevent and quickly correct errors. Within an ongoing musical context, elements should be continuously compared with preceding and upcoming ones, and incongruous events -with respect to the musical syntax and score- should be detected and corrected rapidly. WM function enables such comparisons within an ongoing context. Both Experts and Amateurs regularly train and thus enhance their WM function, yet Experts do so much more extensively, and even more so because stage performance puts an extra strain on WM. This may explain why only differences between Experts and Non-musicians reached significance, although the scores of Amateurs were situated in between those of Non-musicians and Experts.

### ERP waveform results

The waveforms at Fz and Cz shown in Figure 3 show enhanced P3b-like positive voltages with increasing expertise between 300 and 500 ms after stimulus onset in response to the transgressions in particular. The P3b is a subcomponent of the P300 that is involved in context-based memory updating, or “template-matching” (Kok, 2001; Polich and Criado, 2006).

The voltage patterns as a function of expertise, resemble to some extent the pattern of the d-prime values of the musical test. Overlapping curves for Amateurs and Experts occurred in response to T^app^ at Cz, which completely dissociate from the Non-musician group. For T^sub^, we can observe the opposite effect, with differences showing between Experts and the 2 non-expert groups at Fz, the latter of which practically overlap. Therefore, we again observe a 2-by-2 grouping of values in which Non-musicians and Amateurs are associated for T^sub^, and Experts and Amateurs are associated for T^app^. However, all contrasts between expertise levels were significant for the d-prime scores, which is not the case for the waveforms.

The absence of an ERAN-like (Koelsch et al., 2001, 2007) early negativity in response to the transgressions may be explained by the refined in-key incongruities used here, with an absence of any marked dissonance or other sensory cue marking the transgressions (Poulin-Charronnat et al., 2006; James et al., 2008, 2015). Our conclusion of the absence of an ERAN is based on the lack of any kind of negative voltage deflection in response to the transgressions in any group around 200 ms (Koelsch et al., 2007). Other studies using more salient violations first observed an ERAN, then a P3b in non-musicians and musicians (Koelsch et al., 2007; Carrion and Bly, 2008).

Finally, the most essential periods of significant micro-volt differences between the groups (~300–500 ms) coincide with the period retained for the microstate analyses, indicating that these observations are not bound to Fz and Cz, but represent a general mechanism involving the whole scalp topography.

### Microstate results

The statistical testing of the microstates (fitting procedure, see Figure 5) confirmed that Microstate 4, a frontal negativity with right hemisphere dominance, characterized Non-musicians' processing. Given that the Non-musician group spent almost no time in microstate 5 or 7 and spent the same moderate amount of time as the Experts group in Microstate 6, these naïve listeners seem to adopt an intrinsically different way of processing the here used musical stimuli between 300 and 500 ms. In written testimonies that were requested after participants passed the musical test, the Non-musician participants expressed themselves using affective terms to describe the differences they experienced between satisfactory and non-satisfactory closures, using negative terminology for the transgressions. As the Non-musician group had relatively low d-prime scores on average for the T^sub^ stimuli, which they considered fairly “correct,” these testimonies concern mainly the T^app^ stimuli. Apparently, Non-musicians responded to these stimuli based on subjective feeling. In contrast, Expert participants used technical terms related to music theory. Most of them could exactly describe and identify both manipulations used (T^sub^ and T^app^) and even evoke specific stimuli. Amateurs held an intermediate position for Microstate 4, but not for the apparent transgressions. As in the behavioral results, measures for Amateurs and Experts were quite close for T^app^.

Microstates 5 and 7, which were centro-parietal positivities, manifested longer durations with increasing expertise. They show an extended P300/P3b like voltage configuration with more posterior positivity for Microstate 7, likely reflecting top-down cognitive processing, during which the expert listener compares the previously perceived closure with an internal model (Kok, 2001; Polich and Criado, 2006; Carrion and Bly, 2008). The phenomenon of progressively more posterior positivity over time -Microstate 7 appeared on average after Microstate 5 (cf. Figure 4)- in musicians in response to similar transgressions in piano pieces was previously observed in a study comparing expert pianists to non-musicians in a similar time window (see Figure 3 in James et al., 2008).

### Electrical source imaging results

#### Expertise × transgression interaction

The peakvoxel of interaction between expertise and transgression levels occurred in the right posterior middle temporal gyrus (rMTG) in BA 37. However, as observed for d-prime values, ERP and microstate results, Experts manifested higher (CD) than Non-musicians and Amateurs in response to T^sub^, and Experts and Amateurs both displayed higher CD than Non-musicians in response to T^app^. This secondary auditory area is most likely involved in “sound-to-meaning-mapping” in language processing, operated via a connection from the posterior left MTG (lMTG) to the left inferior frontal areas via the ventral pathway (Hickok and Poeppel, 2004). The posterior MTG seems to function as an interface between the sound representations in the superior temporal gyrus and more abstract representations, which are largely distributed in the brain (Hickok and Poeppel, 2004). The lMTG can thus be considered the “gateway” to this processing chain in language processing. We recently showed the existence of a right hemisphere homologue of this white matter pathway for music processing in the same group of participants. The morphology of this pathway became gradually more concise as a function of expertise level, and this increased precision or “Tract Consistency” of the pathway correlated with higher d-prime values for T^sub^ and T^app^ (Figure 3E in Oechslin et al., 2017). Such an analysis of the meaning of sound patterns seems most pertinent in the context of transgressed musical closure, and may be progressively enhanced following musical training. On the functional level, this connection between the right middle temporal gyrus and right inferior frontal areas via the ventral pathway, specifically implicated in the analysis of auditorily presented stimuli that transgress musical structure, has also been observed (Bianco et al., 2016).

Our preceding fMRI study on the same population (Oechslin et al., 2013b) also gave rise to an Expertise x Transgression interaction within the rMTG, although more anteriorly located. Even though the results cannot be directly compared, as we performed fMRI contrasts that subtracted brain regions that responded to R endings from areas responding to T^sub^ and T^app^, we nevertheless consider this compatible finding interesting. Indeed, identical groups and stimuli gave rise to an interaction effect with the peakvoxel in the rMTG in both fMRI and EEG settings, albeit at different locations. The peakvoxel found in the current experiment is situated in the vicinity of the angular gyrus and the temporal–parietal–occipital junction, and represents a good candidate for a sound-meaning interface (Hickok and Poeppel, 2000). Damage to the posterior MTG has been associated to pitch amusia in non-recovered lesion patients when occurring in the right hemisphere (Sihvonen et al., 2016), and to alexia and agraphia when occurring in the left hemisphere (Sakurai et al., 2008).

Music-syntactic processing, which relies on fine-grained pitch processing, is known to take place mostly in the right hemisphere (Zatorre, 2001; James et al., 2008; Cha et al., 2016; Oechslin et al., 2017).

Microstates 5 and 7, which are characterized by expert processing and context-based memory updating, may rely partially on the right MTG in this musical syntax processing paradigm. The P300/P3b components appear to have temporal-parietal origins as well as anterior cingulate and hippocampal sources (Polich and Criado, 2006; Polich, 2007).

#### Main effect of expertise

A main effect of expertise manifested in three different brain areas, systematically separating responses from musicians (both Experts and Amateurs) from Non-musicians. Musicians always showed substantially higher CD in these areas than Non-musicians. The fact that the three transgression conditions set intrinsically different requirements to the participants may explain why no significant differences occurred between Experts and Amateurs.

Detecting T^app^ implies perceiving an explicit syntactic transgression of correct closure, as it presents a degree IV (Subdominant) instead of a degree I (Tonic). In contrast, T^sub^ provides the proper degree; i.e., all the notes of the appropriate tonic chord are present, only with the third in the bass instead of the root. Only experts recognized this very subtle transgression consistently. As observed for the behavioral, ERP, and microstate results, T^sub^ responses most strongly dissociated Experts from Non-musicians and Amateurs, and T^app^ responses distinguished Experts & Amateurs from Non-musicians. It is therefore plausible that gradual differences between Amateurs and Experts did not appear for the main effect of expertise when all transgression conditions were collapsed. The observed increased CD rather shows a general effect of musical training and proficiency (for all levels of transgression), annihilating differences between Experts and Amateurs, which were primarily expressed for T^sub^.

The bilateral anterior cingulate cortex (ACC; see Figure 6B and Table 3), that showed stronger CD in musicians, plays a role in executive functioning related to conflict monitoring and error detection (Bush et al., 2000; Iannaccone et al., 2015). It seems logical that trained pianists, who cope constantly with error detection and prevention during musical training, would develop a stronger conflict monitoring function. However, dorsal and rostral parts of the ACC appear differently involved the respective cognitive and emotional processes accompanying conflict monitoring and error detection, although a functional continuum between cognitive and affective processing is suggested rather than fully segregated operations (Mohanty et al., 2007). As mainly the rostral but also the dorsal parts of the ACC are present in the ACC cluster identified by our analyses, both cognitive and affective aspects may play a role in the processing of correctness of musical closure. Interestingly, the dorsal ACC appears to connect to prefrontal areas, whereas the rostral ACC connects to limbic zones (Mohanty et al., 2007), corresponding to the other two clusters (in the medial frontal cortex and parahippocampal gyrus) that manifested stronger CD in musicians. This supports our hypothesis on the double nature (emotional and cognitive) of closure congruity processing. After all, music represents an emotional stimulus, and deceptive cadences as used here induce -as the terminology suggests- a kind of deception, especially in trained musicians. But such non-conclusive endings also challenge cognitive processing of music. Magno et al. (2006) suggested an evaluative function of the ACC and found the highest ACC activity for error *prevention*.

The adjacent medial frontal gyrus may participate in this function of error monitoring and evaluation on the cognitive and affective levels (Ridderinkhof et al., 2007; Simons, 2010). Moreover, this area seems specifically associated to tonality processing, representing an interface of cognitive, affective, and mnemonic processing (Janata et al., 2002a).

The activations in the right parahippocampal gyrus, which also contains parts of the amygdala and the hippocampus proper, nicely match with earlier findings on expert pianists' responses to apparent in-key harmonic-syntactic incongruities in piano music (peaking between 350 and 400 ms after musical closure onset; James et al., 2008). Apart from the obvious “emotional” hypothesis, these right medial-temporal lobe structures are also involved in higher-order pitch and tonality processing. Indeed, patients with right-sided amygdala-hippocampectomy were severely hampered performing the Seashore musical aptitude test (Wieser, 2003; James et al., 2008), and healthy musically experienced persons processing tonality changes also activated their right hippocampus (Janata et al., 2002b). In the same vein, the amygdala has received the epithet “relevance detector” (Sander et al., 2003), because it seems implicated in the appraisal of relevant events. Now, musical congruity processing is highly relevant and a much-practiced skill for musically trained people.

If we compare the current results with the main effects of expertise in our fMRI study (Oechslin et al., 2013b), the peak value was also found in the right ACC, but in contrast to the current study, only right hemisphere expertise effects were found. However, as mentioned above, as we performed fMRI contrasts that subtracted brain regions that responded to R endings from areas responding to T^sub^ and T^app^, the results cannot be directly compared, and left-sided ACC in musicians may have been cancelled out by the fMRI contrasts. As for the rMTG cluster identified in the Expertise × Transgression interaction, the ACC may also partially underlie Microstates 5 and 7, and may underpin error detection and conflict monitoring, which are closely linked to context-based memory updating. Both error detection and conflict monitoring are necessary to detect an error or deviance with respect to an internal learned model.

#### Main effect of transgression

This experiment focused on gradual brain adaptations as a function of musical expertise and proficiency in the context of different levels of transgression saliency. Therefore, the main effect of Transgression is of the least interest in the current study, as all expertise levels were collapsed. Moreover, as the three transgression conditions set intrinsically different requirements for the participants, as explained in the second paragraph of the preceding subchapter *Main Effect of Expertise*, widely distributed brain clusters responded. Discussing all of them would lead us out of the scope of this study, which principally focuses on musical expertise.

However, the bilateral clusters in the ACC and medial frontal cortex as well as in the parahippocampal gyrus that contributed to the main effect of Transgression pertain to the same brain areas as those representing the main effect of Expertise and showed systematically higher CD in response to T^app^ as compared to R and T^sub^. In all participants, responses to apparent thus more salient transgressed endings evoked stronger responses in these brain areas than regular and subtly transgressed closures, independent of expertise level. As both Non-musicians and Amateurs appraised T^sub^ with relatively low d-prime values, the accurate appraisal by Experts seems to be annihilated by the collapsing of the groups. The explanations we provided for the involvement of the ACC, medial frontal cortex, and parahippocampal gyrus for the factor Expertise also hold for the Transgression condition without taking Expertise into account: all participants -to varying degrees- were involved in error monitoring and evaluation on the cognitive and affective levels and performed higher-order pitch analyses. However, it seems that when all expertise levels are collapsed, more involvement of d-ACC and thus the cognitive component of the error monitoring manifests for the main effect of transgression discussed in this subchapter than for the main effect of expertise (see Figure 6Ciii). This may be explained by the fact that the task becomes progressively easier with increasing expertise, demanding less cognitive effort and likewise, requiring less error prevention.

The stronger activations in the infero-frontal regions, close to the Broca area, can be explained by their participation in musical syntax processing (Maess et al., 2001; Zatorre, 2001; Levitin and Menon, 2003; Tillmann et al., 2006; James et al., 2008; Koelsch et al., 2013) as well as in working memory function (Janata et al., 2002b; Oechslin et al., 2017). Both explanations are plausible in the context of detecting transgressions while appraising the endings of rich polyphonic musical pieces.

Comparing the current results with the main effects of transgression in our fMRI study (Oechslin et al., 2013b), no overlapping activations occurred. Again, apart from the different neural mechanisms involved, this may be due to the fMRI contrasts used, specifically for the effect of transgression, as we subtracted brain regions that responded to R endings from areas responding to T^sub^ and T^app^.

### Limitations of the study

In the first place, as we only investigated classically trained pianists, all our conclusions must therefore be restrained to this subpopulation of musicians. This has some impact because pianists who perform the classical repertoire, reading and performing complex polyphonic scores, may show higher proficiency in syntax processing than musicians playing melodic instruments like the violin or the flute.

In addition, although electrical source imaging methods using spherical head models and template anatomical images similar to ours have been successfully applied in different cognitive and clinical domains (James et al., 2008, 2015; Britz et al., 2009, 2014; Corrigan et al., 2009; Knebel et al., 2011; Geukes et al., 2013; Plomp et al., 2013; Rihs et al., 2013; De Meo et al., 2015), they can only guarantee centimeter spatial accuracy (Plomp et al., 2010).

Finally, as we did not use digitized electrode positions, or individual T1 anatomical images, we lose around 10% in sensitivity of source localization (Brodbeck et al., 2011) compared to recent studies that applied these individualized approaches (Birot et al., 2014; Megevand et al., 2014; Plomp et al., 2015).

## Conclusion

The main conclusion resides in the confirmation of gradual impact of musical training on brain and behavior, which is now for the first time also supported by EEG methods with millisecond precision.

In a time window from approximately 300–500 ms, non-musicians, amateur musicians and professional musicians responded differently to realistic complex musical stimuli at the behavioral, ERP, microstate, and brain source levels. With increasing expertise, enhanced processing proficiency allowed comparing the previously perceived musical closure with a learned internal model, implicating top-down cognitive processing, as evidenced by extended P300/P3b-like configurations, that expressed on the waveform and the microstate level, supported by the posterior rMTG, bilateral ACC and right parahippocampus. In addition, the right middle temporal gyrus may represent the gateway of sound-to-meaning-mapping, a processing chain in music processing from the rMTG toward frontal areas via the ventral stream.

Methodologically, it is compelling to note that peakvoxels for the main effect of Expertise (ACC) and Expertise × Transgression interaction (rMTG) were situated in the same brain areas, albeit at different locations, when EEG source imaging data and fMRI were applied and identical stimuli and participants were examined.

Given the strict matching of the 3 expertise groups, these results provide evidence on the progressive impact of training on brain and behavior.

## Author contributions

CEJ conceived the study, performed the data collection together with MSO, realized a first draft of all analyses, conceived the outlines of all figures and wrote and edited the manuscript. MSO performed the data collection together with CEJ, and contributed to the preprocessing of the data. Then he contributed importantly to the final editing of the manuscript. He was involved in all other studies pertaining to Swiss National Science Foundation (100014–12505 to CEJ, see Funding). CMM participated by means of his expertise in the microstate and source analyses, and also performed a depth reading of the manuscript. MD realized the final ERP analyses and figures and contributed importantly to the final editing of the manuscript.

### Conflict of interest statement

The authors declare that the research was conducted in the absence of any commercial or financial relationships that could be construed as a potential conflict of interest.

## Footnotes

^1^Harmony refers to the rule system of chord structure in Western music.
