# Supplementary material for: Electrical Neuroimaging of Music Processing Reveals Mid-Latency Changes with Level of Musical Expertise
Source: Front Neurosci. 2017 Nov 7;11:613. doi: 10.3389/fnins.2017.00613 (PMC5682036; doi:10.3389/fnins.2017.00613)
Supplement: Supplementary file 1 [file Table1.docx]

**Supplementary Table 1**

Time periods of Bonferroni corrected significant differences between N, A and E for each electrode and each Transgression condition with a minimum time constraint of 28 consecutive ms.

|  |  | Period 1 [ms] | Period 2  [ms] | Period 3  [ms] |
| --- | --- | --- | --- | --- |
| **REGULAR** | |  |  |  |
| Cz | E vs N | 288-520 | 556-668 | - |
|  | E vs A | - | - | - |
|  | A vs N | 288-316 | 384-508 | 556-596 |
| Fz | E vs N | 184-212 | 284-488 | - |
|  | E vs A | 332-432 | - | - |
|  | A vs N | - | - | - |
|  |  |  |  |  |
| **SUBTLE TRANSGRESSION** | |  |  |  |
| Cz | E vs N | 404-576 | 584-636 | - |
|  | E vs A | - | - | - |
|  | A vs N | - | - | - |
| Fz | E vs N | 284-460 | - | - |
|  | E vs A | 324-444 | - | - |
|  | A vs N | - | - | - |
|  |  |  |  |  |
| **APPARENT TRANSGRESSION** | |  |  |  |
| Cz | E vs N | 252-684 | - | - |
|  | E vs A | - | - | - |
|  | A vs N | 260-600 | 612-664 | - |
| Fz | E vs N | 12-56 | 212-292 | 308-388 |
|  | E vs A | - | - | - |
|  | A vs N | 324-388 | - | - |
